# Retraction: The generation and characterization of novel *Col1a1*^*FRT-Cre-ER-T2-FRT*^ and *Col1a1*^*FRT-STOP-FRT-Cre-ER-T2*^ mice for sequential mutagenesis

**DOI:** 10.1242/dmm.028340

**Published:** 2016-12-01

**Authors:** Minsi Zhang, David G. Kirsch

Retraction of: *Dis. Model. Mech.* **8**, 1155-1166.

The authors write:

In this paper, we reported two strains of novel mice: *Col1a1*^*FRT-STOP-FRT-Cre-ER-T2*^ and *Col1a1*^*FRT-Cre-ER-T2-FRT*^. For figure 7, we generated primary sarcomas in *Col1a1*^*FRT-STOP-FRT-Cre-ER-T2*^*; Kras*^*FRT-STOP-FRT-G12D/+*^*; p53*^*FRT/FRT*^*; Rosa26*^*mTmG/+*^ mice with intramuscular Adeno-FlpO and attempted to activate eGFP expression in tumors by injecting the mice with intraperitoneal (IP) tamoxifen. We observed that, following conventional IP tamoxifen administration, the tumors did not express eGFP. We also injected sarcomas directly with 4-hydroxy-tamoxifen (4-OHT). After multiple doses of 4-OHT injected directly into the tumor, we noted varying degrees of eGFP expression. Based on these results, we concluded that the low recombination efficiency of the *mTmG* allele *in vivo* after IP injection was due to limited penetration of the tamoxifen metabolite into the tumor in this sarcoma model.

Members of our lab have performed additional characterization of *Col1a1*^*FRT-Cre-ER-T2-FRT*^ and *Col1a1*^*FRT-STOP-FRT-Cre-ER-T2*^ mice. Although the description of our characterization of the *Col1a1*^*FRT-Cre-ER-T2-FRT*^ allele remains valid, additional characterization of FlpO-activated sarcoma cell lines and mouse embryo fibroblasts (MEFs) from the *Col1a1*^*FRT-STOP-FRT-Cre-ER-T2*^ mice show inefficient recombination of FLOX alleles when exposed to 4-OHT *in vitro*. This seems to be due to low expression of CreER following activation by FlpO recombinase. These new results indicate that our original conclusion that the poor uptake of tamoxifen by sarcomas impaired recombination from the FlpO-activated CreER in *Col1a1*^*FRT-STOP-FRT-Cre-ER-T2*^*; Kras*^*FRT-STOP-FRT-G12D/+*^*; p53*^*FRT/FRT*^*; Rosa26*^*mTmG/+*^ mice is incorrect. Instead, low expression of CreER after FlpO activation in *Col1a1*^*FRT-STOP-FRT-Cre-ER-T2*^ mice likely contributed to this phenotype. We apologize to readers for this error. Because the follow-up experiments using FlpO-activated cells derived from *Col1a1*^*FRT-STOP-FRT-Cre-ER-T2*^ mice do not show efficient recombination of FLOX alleles *in vitro*, we would like to retract our paper.

